# The complete mitogenome of *Lycostomus* sp. (Elateroidea: Lycidae)

**DOI:** 10.1080/23802359.2019.1682483

**Published:** 2019-10-26

**Authors:** Hao-Yu Liu, Zi-Xuan Kang, Fang Zhang, Xue-Ying Ge, Yu-Xia Yang

**Affiliations:** The Key Laboratory of Zoological Systematics and Application, College of Life Sciences, Hebei University, Baoding, Hebei Province, China

**Keywords:** Mitochondrial genome, *Lycostomus* sp., Lycidae

## Abstract

The complete mitochondrial genome of a net-winged beetle was sequenced, *Lycostomus* sp. (Coleoptera: Lycidae). The total length of this mitogenome is 16096 bp and the composition of each base is A (41.1%), T (31.9%), C (17.1%), G (9.9%), respectively. The gene arrangement of this beetle mt genome is the same as other insects. The phylogenetic tree shows that *Lycostomus* sp. is closest to *Platerodrilus* sp. with robust statistical support, which confirms the monophyly of Lycidae.

The genus *Lycostomus* Motschulsky, 1861 belongs to the family Lycidae Laporte, 1836. Over one hundred species are included in this genus and widely distributed in the Oriental and Holoarctic regions (Kleine 1933; Bocákova and Bocák 2007). The adults could be easily differentiated from others by the elongate head which prolonged before the eyes to form a rostrum probably to facilitate feeding on deep-seated nectaria (Kazantsev 2005). Besides, pronotum is present with median longitudinal carina in anterior part, pronotal margins are explanate along the edge; elytra are slightly to moderately widened posteriorly, each elytron has four longitudinal costae, but recticulate costae are reduced, irregular; male genitalia is equipped with slender aedeagus, short paramere, which is rarely reaching half of the phallic length; female genitalia has extensive plate-like coxites and separated and slender paraproctal baculi (Bocák and Bocákova 2008). Up to now, only one complete mitogenome of lycid species is available at GenBank.

Here, the second complete mitochondrial genome for Lycidae was sequenced with *Lycostomus* sp. chosen as the representative. The specimens used in this study were collected from Hongping Forestry, 31°36′24″N, 110°25′02″E, Shennongjia, Hubei Province, China and deposited in the Museum of Hebei University, Baoding, China (MHBU, accession number CAN0014). Genomic DNA was extracted by DNeasy Blood & Tissue kit (QIAGEN, Germany). Illumina TruSeq libraries were prepared using genomic DNA with an average insert size of 450 bp and were sequenced on the Illumina Hiseq2500 platform with 250 bp paired-end reads at BerryGenomics (Beijing, China). The sequence reads were first filtered by the programs following Zhou et al. (2013) and then the remaining high-quality reads were assembled using IDBA-UD (Yu and Henry 2012). In order to study the accuracy of assembly, Geneious 2019.2 was used to map clean reading onto the mt genome sequence. The annotations of genes were done by Geneious 2019.2 software and tRNAscan-SE 1.21 (Schattner et al. 2005). The annotated sequence was registered in GenBank with accession number MN264644.

Like most animals (Gissi et al. 2008), the mitogenome of *Lycostomus* sp. encodes for 37 genes. The structure and compositions of the mitogenome are similar to *Platerodrilus* sp. (Lycidae) and other closely related beetles (e.g. Sheffield et al. 2008). It is a double-stranded circular molecule of 16,096 bp in length, which contains 22 tRNA genes, 13 protein-coding genes (PCGs), 2 rRNA subunits, and an AT-rich region as in other insects. The composition of each base was calculated as A (41.4%), T (31.9%), C (17.1%), G (9.9%), and GC content was 27%. The overall A + T composition was relatively high (73%), as *Platerodrilus* sp. (76.2%) and other species are included in Elateroidea. The start codon of ND1 was abnormal TTG. ATN codon was used in other 12 PCGs. TAA or TAG was used as terminal codon, except an incomplete terminal codon, namely AA was found in *COX2, COX3, ND4,* and *ND5*.

The newly determined mitogenome sequence was aligned by the Tran Align methods (Bininda-Emonds 2005) with orthologous sequences corresponding to complete mitogenomes available in GenBank for Elateroidea (13 species) and closely related Dryopidae (1 species) and Buprestidae (1 species) as outgroup. These species were as follows: *Chauliognathus opacus* (Sheffield et al. 2009), *Platerodrilus* sp. (Uribe and Gutiérrez-Rodríguez 2016), *Rhagophthalmus ohbai* (Li et al. 2007), *Brasilocerus* sp., *Hapsodrilus ignifer*and *Bicellonychia lividipennis* and *Hapsodrilus ignifer* (Amaral et al. 2016), *Melanotus villosus* (Linard et al. 2016), *Anostirus castaneus*, *Adrastus rachifer*, and *Lampyris noctiluca* (Linard et al. 2018), *Aquatica leii* (Jiao et al. 2013), *Limonius californicus* (Gerritsen et al. 2016), Cerophtidae sp., *Dryops ernesti* and *Chrysochroa fulgidissima* (Hong et al. 2009). The aligned data were concatenated with Sequence Matrix v.1.7.8 (Vaidya et al. 2011). Data were partitioned according to loci of 13 PCGs. The neighbor-joining tree was constructed by MEGA 7.0 with 1500 bootstrap replicates, based on Kimure-2 parameter model. The bootstrap showed sufficient value at all nodes. The reconstructed phylogenetic tree shows that *Lycostomus* sp. is closest to *Platerodrilus* sp. with robust statistical support (Figure 1), which suggests that Lycidae is recovered as a monophyletic group. However, within the superfamily Elateroidea, the phylogenetic relationships among families remain unresolved as in other studies (e.g. Timmermans et al. 2016).

**Figure 1.**
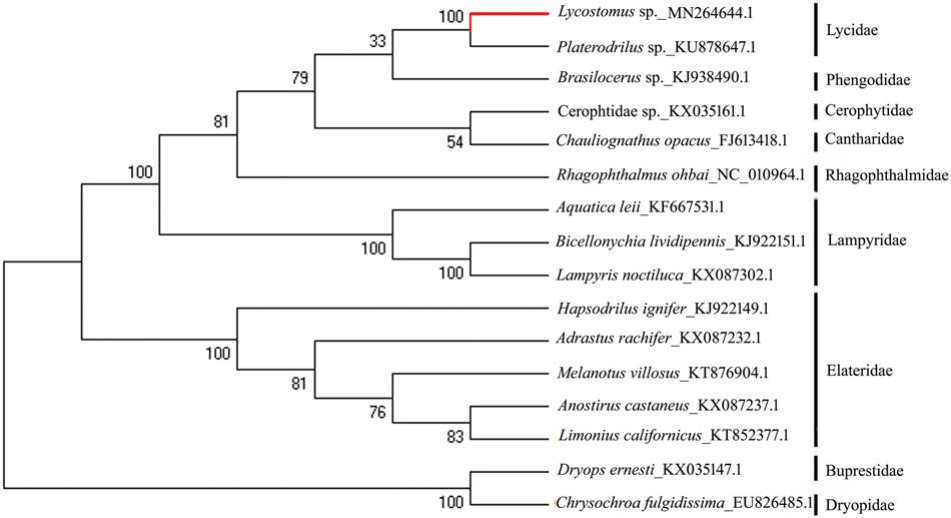
The phylogenetic tree of 16 species of Elateroidea, Dryopidae, and Buprestidae based on 13 PCGs of mitochondrial genome sequence.
